# Evidence from Outcomes: Gender-Neutral 2vHPV Vaccination at Moderate Coverage Drives Rapid Depletion of HPV16/18 Among Vaccinated and Unvaccinated Women

**DOI:** 10.3390/v18010099

**Published:** 2026-01-12

**Authors:** Matti Lehtinen, Ville N. Pimenoff, Tiina Eriksson, Camilla Lagheden, Anna Söderlund-Strand, Heljä-Marja Surcel, Joakim Dillner

**Affiliations:** 1 Center for Cervical Cancer Elimination, Karolinska Institutet, 171 77 Stockholm, Sweden; matti.antero.lehtinen@gmail.com (M.L.); joakim.dillner@ki.se (J.D.); 2 Finnish Institute of Health and Welfare, P.O. Box 30, FI-00271 Helsinki, Finland; 3 Biobank Borealis of Northern Finland, University of Oulu, P.O. Box 8000, FI-90014 Oulu, Finland; 4 Unit of Population Health, University of Oulu, P.O. Box 8000, FI-90014 Oulu, Finland; 5 Faculty of Medicine, Tampere University, FI-33014 Tampere, Finland; 6 Department of Laboratory Medicine, Clinical Microbiology, Skåne University Hospital Lund, Lund University, Box 117, 221 00 Lund, Sweden

**Keywords:** gender-neutral, HPV vaccination, community randomized, HPV genotyping, serology, HPV eradication

## Abstract

Human papillomavirus (HPV) vaccination may eventually eradicate oncogenic vaccine-targeted HPVs but only with a strategy that also protects unvaccinated individuals. We compared the impact of gender-neutral and girls-only vaccination strategies on the indirect and direct protection of unvaccinated and vaccinated young women against HPV16/18 infection using HPV16/18 seropositivity and PCR positivity 3–7 years post vaccination as the outcome measure. A total of 33 Finnish communities were randomized to one of three vaccination strategies: bivalent gender-neutral HPV vaccination (Arm A), girls-only HPV vaccination (Arm B), or control hepatitis B vaccination (Arm C). All individuals born between 1992 and 1995 and residing in these communities (n = 80,272) were invited to participate. Overall, 11,662 males and 20,513 females consented, corresponding to vaccination coverages of 25% and 45%, respectively, in 2007–2009. Between 2010 and 2014, 11,396 cervical samples were collected from 18-year-old participants and subjected to high-throughput PCR-based HPV genotyping. In addition, serum samples were obtained from 8022 unvaccinated women under 23 years of age residing in Arm A (n = 2657), Arm B (n = 2691), or Arm C (n = 2674) communities during the pre-vaccination (2005–2010) and post-vaccination (2011–2016) periods. To assess indirect vaccine effects using PCR and serological outcomes in unvaccinated women, we compared reductions in HPV16/18 prevalence from baseline within the gender-neutral and girls-only vaccination arms, using the control arm as a reference. A significant decrease in seroprevalence between the pre- and post-vaccination periods was detected in the gender-neutral communities for both HPV16 (seroprevalence ratio = 0.64) and HPV18 (0.72), whereas no comparable reductions were observed in the girls-only or control communities. In contrast, a significant reduction in HPV18 PCR-based prevalence from baseline to the post-vaccination period was observed in both the gender-neutral (0.32) and girls-only (0.61) communities. However, after accounting for ratios of seroprevalence rations for secular trends, the corresponding decrease in HPV18 seroprevalence was no longer statistically significant. Vaccine efficacy (VE) in Arm A or Arm B versus Arm C of vaccinated women measured the direct protection of vaccinated women by vaccination strategy. HPV16/18 VEs varied between 89% and 96% with some indication of herd effect against HPV18. Robust effectiveness of vaccination against PCR-confirmed cervical HPV16/18 infections, along with rapid indirect protection against HPV16/18 and HPV18 infections, was evident even with vaccination reaching only 25% and 45% coverage. Our results suggest that vaccine efficacy and herd effect induced by gender-neutral 2vHPV vaccination sets the stage for comprehensive HPV eradication, including the unvaccinated in the vaccinated communities.

## 1. Introduction

Human papillomavirus (HPV) associated anogenital cancers and their precursor lesions can be efficiently prevented by prophylactic vaccination [1,2,3]. Registry-based long-term follow-up of clinical phase III trials on both the bivalent and the quadrivalent vaccine [4] have shown that this protection is sustainable at least for 15 years [5,6,7]. The school-based vaccination programs have a HPV vaccination coverage that at best ranges between 75% and 95% among early adolescent females [8]. However, with very few exceptions, the HPV vaccination coverage is not higher than 50% in the affluent countries and is even lower in the low and middle income countries [8]. This leaves notable proportions of susceptible adolescent females entering sexually active life without protection against oncogenic high-risk (hr) HPV types [9].

Based on our community-randomized trial data we have advocated for the superior impact of gender-neutral HPV vaccination in establishing herd effect (HE, indirect protection) to protect also the unvaccinated individuals on top of providing at least the same direct vaccine efficacy (VE) to those adolescent females who get vaccinated [9]. Together with the rapid reduction in background hrHPV prevalence, the HE and VE provide the ultimate overall protective effectiveness (PE) to the entire population of young individuals [10,11,12]. Initial results of our community-randomized trial suggested that to establish sufficient herd effect against HPV type 16 would require much higher than 50% vaccination coverage also from the gender-neutral strategy. However, by defining the outcome as persistent HPV infection by seropositivity [13], rather than mere one-time PCR positivity, we could show that the gender-neutral vaccination generated significant herd effect, as measured by HPV seroprevalence reduction in the unvaccinated females, both against HPV16 and HPV18 [14].

In this study we have now compared for the first time the impact of gender-neutral and girls-only HPV vaccination strategies against incident HPV16/18 infections using HPV PCR positivity and persistent HPV16/18 infection using HPV seropositivity as an end-point.

## 2. Materials and Methods

### 2.1. Intervention

For this community-randomized study (NCT00534638), 33 Finnish communities were stratified by HPV16/18 seroprevalence in female Finnish Maternity Cohort participants under 23 years of age during 1983–2003 into equally sized strata of low (<20.5%), intermediate (20.5–24%), and high (>24%) seroprevalence. The randomization in each strata was to one of three factorial trial arms; intervention Arm A or B and control Arm C [15].

**Figure 1:**
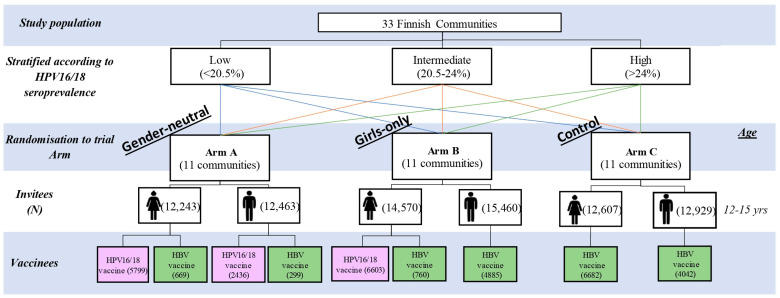
Trial design [10,11,12].

In 2007–2009, a total of 80,272 Finnish or Swedish speaking girls and boys born between 1992 and 1995 and resident in the 33 communities were invited to participate at the age of 12–15 years (Figure 1). A total of 32,175 individuals participated in the study with parental/guardian informed consent. Within the trial arms, Arm A received gender-neutral vaccination, with 90% of participating girls and boys receiving the bivalent HPV16/18 vaccine (2vHPV) and 10% the hepatitis B-virus vaccine, Arm B received gender-specific vaccination with 90% of female participants receiving HPV vaccination and 10% HBV vaccination whilst all males participants received the HBV vaccine, and Arm C received gender-neutral HBV vaccination. Of all the participants, 99.4% received three doses of the vaccine in a 0-, 1-, and 6-month regimen [15]. 

In Arm A communities, birth cohort-wise vaccination coverage was 30.5% (standard deviation, SD = 7.8), 30.9% (SD = 7.0), 36.3% (SD = 7.8), and 35.5% (SD = 7.9) amongst all those born, respectively, in 1992, 1993, 1994, and 1995. In Arm B communities, the birth cohort-wise vaccination coverage was 22.0% (SD = 2.1), 21.2% (SD = 3.7), 23.3% (SD = 3.7), and 22.1% (SD = 3.1) amongst all those born, respectively, in 1992, 1993, 1994, and 1995.

Active follow-up. During years 2010–2014, the female participants were invited to attend a follow-up visit at age 18 when cervical DNA samples were taken. The originally non-HPV-vaccinated participants were also offered HPV cross-vaccination.

Passive follow-up. was through linkage with the Finnish Maternity Cohort (FMC), a population representative cohort comprising over 2 million serum samples from approximately 96% of pregnant women in Finland from 1983 until 2016 [16]. The first-trimester FMC samples were eligible for inclusion into the study population if the women who donated the sample were under the age of 23 years, had donated between 2005 and 2016, and were a resident in one of the 33 study communities at the time of sample donation. All eligible FMC samples/donors were linked to a register of HPV-vaccinated individuals to identify and exclude any individuals who were HPV-vaccinated via either the HPV-040 (NCT00534638), PATRICIA (NCT01393470), FUTURE (NCT00092534), or HPV012 (NCT00337818) studies conducted prior to the start the national HPV vaccination program in 2014.

**Figure 2:**
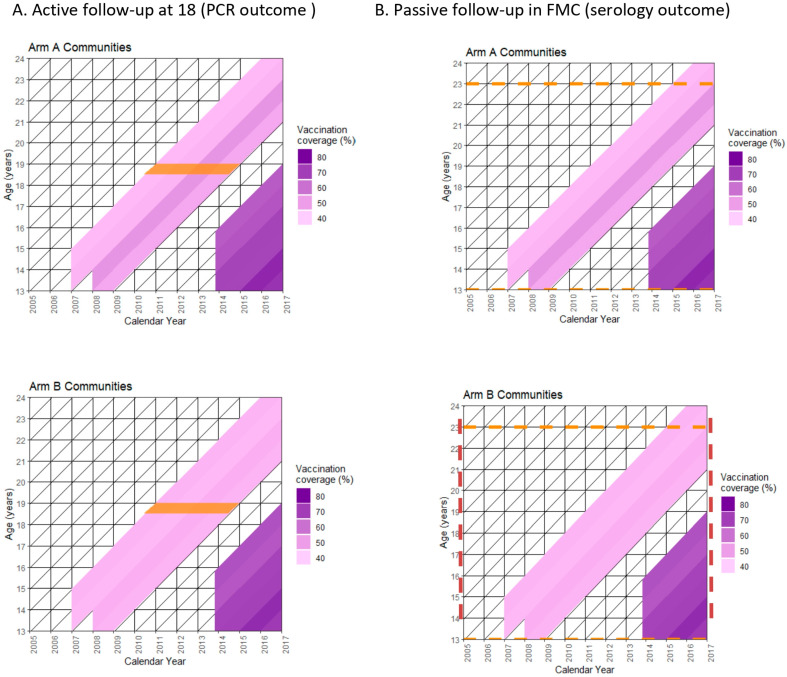
Lexis diagrams showing study populations of the two different follow-up approaches and vaccination coverage among pertinent birth cohorts. The study populations are indicated by orange bar ((**A**) Active follow-up with HPV PCR end-point) or dotted orange lines ((**B**) Passive follow-up with HPV serology end-point).

Sixty eligible samples were selected from each of the 33 trial communities for four consecutive time periods: 2005–07, 2008–10, 2011–13, and 2014–16). If there were more than 60 individuals, a random sample of 60 individuals was selected using SPSS statistical software version 22. For those strata with less than 60 eligible donors, all those eligible were selected. The repeated cross-sectional sampling of samples donated during the calendar years 2005–07 and 2008–10 were representative of the pre-vaccination era, and the two later time periods (2011–13 and 2014–16) were representative of the post-vaccination era. The final cross-sectional cohort provides a measure of cross-exposure in the control Arm C due to cross-vaccination of our community-randomized trial participants, and increased exposure in all three arms owing to the commencement of the HPV national vaccination program plus the catch-up program targeting the 1998 birth cohort and younger with 65 to 75% vaccination coverage among the 1998–2004 birth cohorts [14] (Figure 2).

### 2.2. Laboratory Methods

The serum samples were analyzed for the presence of antibodies to HPV16 and HPV18 and HSV-2 using heparin bound pseudovirion luminex serology as described [17]. The DNA samples were analyzed by PCR for HPV DNA. HPV typing was performed by matrix assisted laser desorption time-of-flight (MALDI-TOF) mass spectrometry for the detection of HPV6/11/16/18/31/33/35/39/45/51/52/56/58/59/66/68 [10,18].

### 2.3. Statistical Analysis

After adjusting for smoking and mobility between communities the prevalence ratios (PR) with 95% CI of HPV types 16, 18, and 16/18 between Arm A and C, and Arm B and C were estimated by log-binomial regression as described [19].

To determine vaccine efficacy in HPV- versus HBV-vaccinated 18 year-old women, 1-RR of HPV infection (HPV DNA positivity) was estimated for Arm A versus Arm C, and Arm B versus Arm C by the Generalized Estimating Equation (GEE) method (binomial response and logit link was applied). An exchangeable correlation structure between responses (HPV results) of women from the same community, and independence between responses of women from different communities were assumed. The 95% confidence intervals (95% CI) were based on profile likelihood [10,11].

### 2.4. Ethics

The community-randomized study obtained permissions from the Ethical Review Board of Pirkanmaa Hospital District (R07113M 14.6.2007). Informed consent to use the FMC serum samples for research purposes was granted by the pregnant women at sample donation.

## 3. Results

PCR-based active follow-up and serology-based passive follow-up of our community-randomized trial cover overlapping birth cohorts of 18 to 19 year-old (1992–1995 born) women and 16 to 22 year-old (1983–2000 born) women over calendar time periods of 2010–2014 (active follow-up) and 2005–2016 (passive follow-up) (Figure 2). HPV vaccination coverage among the intervention targeted females resident in Arm A (gender-neutral) and Arm B (girls-only) communities which varied between 25% and 45% in the community-randomized HPV vaccination trial between 2007 and 2010 and during the gradual start implementation of the Finnish national girls-only HPV vaccination program, which started in 2014 (Figure 2).

As compared to the baseline, significant HPV seroprevalence reduction was observed among the unvaccinated young women both for HPV16 (0.64) and HPV18 (0.72) in the gender-neutral communities but not in the girls-only communities (Table 1). Even if significant baseline vs. post-vaccination HPV18 PCR-prevalence reduction was observed, both in the gender-neutral (0.32) and girls-only (0.61) communities, the latter lost statistical significance if ratios of seroprevalence rations were considered by comparison to the control arm communities (Table 1). No indication of HPV16 PCR-prevalence reduction was observed in the overall study population.

HPV vaccine efficacy (VE) in Arm A or Arm B versus control Arm C (HBV-vaccinated women) could be measured using single HPV PCR positivity outcome only. With the former measure, both the HPV16 and HPV18 VEs were all high, between 89 and 96% (Table 2). For the HPV18 vaccine efficacies derived from both the gender-neutral Arm A and girls-only Arm B, we noted somewhat increased VE estimates exclusively around 96% (Table 2).

## 4. Discussion

Our most important finding was a significant reduction in HPV16 and HPV18 seroprevalence observed among unvaccinated adolescent and young adult women during passive follow-up, but only in communities implementing the gender-neutral vaccination strategy. Similarly, a significant decrease in HPV18 PCR-based prevalence between baseline and the end of the study was detected among unvaccinated 18-year-old women exclusively in the gender-neutral communities. Establishing herd effect against HPV16, which has a uniquely high basic reproduction number [20,21], by girls-only vaccination has been reported to require very high, 90%, vaccination coverage [22]. However, we recently documented that with the gender-neutral HPV vaccination strategy, herd effects against both HPV16 and HPV18, as measured with type-specific pre- and post-vaccination seroprevalence reductions, are established already with 25% to 50% vaccination coverage [14]. We now demonstrate that reduction in HPV18 PCR-prevalence, but not HPV16 PCR-reduction, is in line with the seroprevalence reduction results and is valid even after considering secular/temporal changes in the prevalence trends following comparison to the control communities.

The rationale for significant prevalence reduction in HPV18 but not for HPV16 among the unvaccinated adolescent and young adult women via herd effect generated by the gender-neutral strategy are many. Both high transmissibility and slow clearance rates [1,23,24,25] of HPV16 contribute to prevalence fluctuations measured by PCR. Hence, it is difficult to estimate the contribution of herd effect by PCR for the prevalence reduction among unvaccinated women. In contrast, seroprevalence of HPV16 (and HPV18) which is a measure of cumulative incidence of persistent HPV16 (and HPV18) infection, is likely a more robust measure for the herd effect induced prevalence reductions.

Population-based, albeit community-randomized interventions, randomization and the fact that the active follow-up and passive follow-up periods considerably overlapped are strengths of our study.

Due to limitations of the VLP serology, HPV infection and HPV vaccination-induced antibodies could have been distinguished only by differences in the antibody levels which would have been cumbersome here. Thus, we were not able to measure direct protection, i.e., vaccine efficacy by the PCR outcome only. Even if, thereby, PCR-defined HPV vaccine efficacies were high, the highest VEs were observed against HPV18 both in the gender-neutral and girls-only arms, which suggests that herd effect against HPV18 adds to the direct HPV18 vaccine efficacy.

In conclusion, the strong vaccine efficacy against PCR-confirmed cervical HPV16/18 infection, together with the rapid indirect (herd) protection against HPV16/18 infections detected by both PCR and serology in unvaccinated women, even at vaccination coverage levels of 25% and 45%, is clear. These findings highlight that combined vaccine efficacy and herd effect induced by gender-neutral 2vHPV vaccination sets the public health strategy for comprehensive HPV eradication, including the unvaccinated in the vaccinated communities [9,10,11,12].

## Figures and Tables

**Table 1:** (**A**) Post-vaccination versus baseline HPV seroprevalence ratios (sPR) and ratios of seroprevalence ratios (sRPR) among unvaccinated females aged under 23 (2011–2016) and (**B**) post-vaccination versus baseline HPV PCR-prevalence ratios (PR) and ratios of prevalence ratios (RPR) among unvaccinated females aged 18 to 19 year of age (2014).

		Arm A		Arm B			Arm C
A	HPV type	sPR	sRPR	sPR	sRPR		sPR
serology	HPV16	0.64 *	0.60 *	1.19	1.04		1.07
	HPV18	0.72 *	0.91	0.89	1.13		0.79
	HPV16/18	0.66 *	0.79	0.92	1.1		0.84
							
		Arm A		Arm B			Arm C
B	HPV type	PR	RPR	PR	RPR		PR
PCR	HPV16	1.16	1.1	1.42	1.35		1.05
	HPV18	0.32 *	0.68 *	0.61 *	1.3		0.47
	HPV16/18	0.87	1.01	1.02	1.19		0.86

Arm A: gender-neutral HPV vaccination, Arm B: girls-only HPV vaccination, Arm C: control HBV vaccination. * Statistically significant (95% confidence interval).

**Table 2:** Prevalence and vaccine efficacy (VE) of HPV16, HPV18, or HPV16 and HPV18 among females aged 18–19 by intervention arm at the start (2010–2011) and at the end (2014) of the vaccination trial.

		Arm A		Arm B			Arm C
time frame	HPV type	Prevalence	VE	Prevalence	VE		Prevalence
2010–2011	HPV16	0.60%	91.7 *	0.60%	92.1 *		7.10%
	HPV18	0.20%	94.5 *	0.30%	91.6 *		3.90%
	HPV16/18	0.80%	93.2 *	0.90%	89.2 *		10.10%
2014	HPV16	0.50%	93.2 *	0.70%	89.8 *		5.90%
	HPV18	0.20%	96.1 *	0.10%	95.7 *		3.70%
	HPV16/18	0.60%	93.8 *	0.80%	91.2 *		8.50%

Arm A: gender-neutral HPV vaccination, Arm B: girls-only HPV vaccination, Arm C: control HBV vaccination and HPV type. * Statistically significant (95% confidence interval).

## Data Availability

Anonymous study data is available from the corresponding author upon reasonable request after approval by DAC. The metadata from this study is available upon request from the DAC and ethical approval via https://etsin.fairdata.fi.
